# Risk Assessment for Preterm Delivery using the Fetal Fibronectin Test Associated with the Measurement of Uterine Cervix Length in Symptomatic Pregnant Women

**DOI:** 10.1055/s-0038-1667185

**Published:** 2018-09

**Authors:** Tadeu Rodriguez de Carvalho Pinheiro Filho, Vanessa Rocha Pessoa, Thaisa de Sousa Lima, Marcela Melo de Castro, José Juvenal Linhares

**Affiliations:** 1 Gynecology and Obstetrics Residency Program, Santa Casa de Misericórdia de Sobral, Sobral, CE, Brazil; 2 Faculty of Medicine, Universidade Federal do Ceará, Sobral, CE, Brazil

**Keywords:** cervical length measurement, fibronectin, pregnancy, preterm labor, risk, medida do comprimento cervical, fibronectina, gravidez, trabalho de parto prematuro, risco

## Abstract

**Objective** To analyze the use of the measurement of uterine cervix length (MUCL) and the fetal fibronectin (fFN) rapid test as predictors of preterm delivery (PTD) in symptomatic pregnant women assisted at the Santa Casa de Misericórdia de Sobral Maternity Hospital.

**Methods** This was a prospective and analytic study involving 53 parturients assisted between September of 2015 and July of 2016; the participants were between 24 and 34 weeks of gestational age (GA) and presented complaints related to preterm labor (PTL) prodromes. Vaginal secretion was collected for fFN testing, and the MUCL was obtained via transvaginal ultrasonography.

**Results** A total of 58.49% of the subjects showed MUCL < 25 mm, and 41.51% were positive in the fFN rapid test. A total of 48 patients were followed-up until their delivery date, and 54.17% resulted in PTL. The relative risk (RR) for PTD in patients with MUCL < 25 mm was 1.83 (*p* = 0.09, 0.99–3.36, 95% confidence interval [CI]), with a mean time before delivery of 2.98 weeks. Based on fFN positive results, the RR was 3.50 (*p* = 0.002, 1.39–8.79, 95%CI) and the mean time until delivery was 1.94 weeks. The RR was 2.70 (*p* = 0.002, 1.08–6.72, 95%CI) when both tests were used. The RR of PTD within 48 hours, and 7 and 14 days were, respectively, 1.30 (*p* = 0.11, 95% CI 1.02–1.67), 1.43 (*p* = 0.12, 95% CI % 0.99–2.06), and 2.03 (*p* = 0.008, 95% CI 1.26–3.27), when based on the MUCL, and 1.75 (*p* = 0.0006, 95% CI 1.20–2.53), 2.88 (*p* = 0.0001, 95% CI, 1.57–5.31), and 3.57 (*p* = 0.0002, 95% CI 1.63–7.81) when based on positive fFN results. The RR at 48 hours and 7 and 14 days considering both tests was 1.74 (*p* = 0.0001, 95% CI 1.14–2.64), 2.22 *(p = 0*.0001, 95% CI 1.22–4.04), and 2.76 (*p* = 0.0002, 95% CI 1.27–5.96), respectively.

**Conclusion** In symptomatic pregnant women, we concluded that the MUCL < 25 mm associated with positive fFN rapid test indicate increased the risk for PTD. Further studies with larger sample sizes could contribute in supporting the results presented in the current study.

## Abstract (pt)

**Resumo:**

**Objetivo** Analisar a utilização da medida do comprimento do colo uterino (MCCU), e do teste da fibronectina fetal (FNf) como preditores do trabalho de parto pré-termo (PPT), em gestantes sintomáticas, atendidas na Maternidade da Santa Casa de Misericórdia de Sobral.

**Métodos** Foi realizado um estudo prospectivo e analítico, envolvendo 53 parturientes atendidas no período de setembro de 2015 a julho de 2016, com idade gestacional (IG) entre 24 e 34 semanas que tiveram queixas relacionadas a pródromos de trabalho de parto prematuro (TPP), sendo realizada coleta de secreção vaginal para FNf e MCCU por via ultrassonográfica transvaginal.

**Resultados** Um total de 58,49% das pacientes tinham MCCU < 25 mm, e 41,51% tiveram teste rápido de fFN positivo. Foi feito o acompanhamento de 48 pacientes, com 54,17% de PPTs. O risco relativo (RR) para PPT com MCCU < 25 mm foi de 1,83 (*p* = 0,09, 0,99–3,36, intervalo de confiança [IC] 95%), com média de tempo até o parto de 2,98 semanas. Para fFN, o RR foi de 3.50 (*p* = 0.002, 1.39–8.79, IC 95%) e a média até o parto foi de 1,94 semanas. Quando os dois testes foram positivos, o RR foi de 2,70 (1,08–6,72). Para a MCCU, o RR para PPT em 48 horas, 7 e 14 dias foram 1,30 (*p* = 0.11, 95% IC 1.02–1.67), 1,43 (*p* = 0.12, 95% CI % 0.99–2.06) e 2,03 (*p* = 0.008, 95% IC 1.26–3.27), respectivamente. Para FNf, em 48 horas, 7 e 14 dias foi de 1,75 (*p* = 0.0006, 95% IC 1.20–2.53, 2,88 (*p* = 0.0001, 95% IC, 1.57–5.31) e 3,57 (*p* = 0.0002, 95% IC 1.63–7.81) respectivamente. Com os dois testes, o RR em 48 horas, 7 e 14 dias foi 1,74 (*p* = 0.0001, 95%IC 1.14–2.64), 2,22 *(p = 0*.0001, 95% IC 1.22–4.04) e 2,76 (*p* = 0.0002, 95% IC 1.27–5.96) respectivamente.

**Conclusão** Em mulheres grávidas sintomáticas, concluímos que a MCCU < 25 mm e o teste rápido de FNf positivo indicam aumento do risco de PPT. Outros estudos com tamanhos de amostra maiores podem contribuir para apoiar os resultados apresentados no presente estudo.

## Introduction

Preterm delivery (PTD), defined as occurring before 37 weeks of gestational age (GA), has an incidence of 11 to 18% of all pregnancies and is the main determinant of neonatal morbidity and mortality.^1^ This rate has remained constant in the past 50 years despite the various advances in medicine, therefore indicating that primary and secondary prevention interventions are occurring inadequately.^2^ ^3^ This rate remains at an average of 9.9% in Brazil.^4^

Preterm delivery is responsible for 75% of the cases of prematurity; the remaining 25% resulting from elective situations, such as preeclampsia, diabetes, fetal distress, and others. The etiology of PTD includes numerous risk factors; however, its mechanism remains uncertain.^5^ Risk factors for PTD include a history of previous PTD, twinning, bleeding during the second half of gestation, infections (chorioamnionitis, pyelonephritis, and asymptomatic bacteriuria), black ethnicity, maternal age under 16 years or above 35 years, smoking, chronic or acute maternal diseases, anemia, uterine malformations, trauma, placenta previa, placental abruption, intrauterine growth retardation, and drug use. Nevertheless, these risk factors occur in only 50% of all PTD cases, and they eventually contribute to the identification of risk pregnancies.^4^ ^5^ ^6^ ^7^ The most important risk factor is a history of prior PTD.^8^ ^9^

Recent studies demonstrate that the incidence of prematurity can be attenuated through the use of the MUCL, evaluated at the end of the second trimester by transvaginal ultrasonography (TVUS), to predict the probability of spontaneous PTD.^10^ ^11^

Another useful test is the evaluation of the presence of fetal fibronectin (fFN) in vaginal secretion. Fetal fibronectin is an adhesive glycoprotein produced by the trophoblast, present in the maternal-fetal interface, which becomes detectable in the first half of the pregnancy and after 35 weeks of GA. This protein will only be present in situations of mechanical or inflammatory alterations due to damage to membranes or placenta between 22 and 35 weeks of GA. The detection of fFN in pregnant women with GA between 22 and 37 weeks can indicate the probability of evolution to spontaneous PTD; this measurement has a high negative predictive value, therefore preventing unnecessary hospitalizations and interventions.^4^ ^6^

Current studies show that the use of the MUCL in combination with the detection of fFN increases the sensitivity in predicting PTD. Iams et al^2^ report very high rates of PTD recurrence (64%) in women with a positive fFN test result and short MUCL (under 25 mm) in a multicenter study with 1,282 asymptomatic pregnant women with a previous history of PTD. In that study, fFN appeared as the most powerful predictor factor for PTD; in women with MUCL above 35 mm, the PTD recurrence rate was 7% when fFN was negative compared with 28% in those with positive fFN test result. These authors emphasize that the importance of these tests is greatest in pregnant women presenting risk factors for PTD. In the case of pregnant women with no history of previous PTD, the risk of birth before 35 weeks of GA was reported as 13% when the fFN test was positive and 8% when MUCL was shorter than 25 mm. Conversely, when there was a history of previous PTD, the probability of a new event was 40% for those with positive fFN and 30% for those with short MUCL.^12^

Hence, this study evaluated the risk of PTD through MUCL and fFN rapid-test results in pregnant women hospitalized at Hospital Maternidade Santa Casa de Misericórdia de Sobral (SCMS, in the Portuguese acronym), with complaints related to preterm labor (PTL) prodromes.

## Methods

This was an analytical, prospective, invasive, and non-interventional study, performed at the SCMS with pregnant women presenting suggestive PTL symptoms, who were hospitalized between September of 2015 and July of 2016. The sample size was calculated for convenience and non-probability sampling.

The study was approved by the respective Ethics Committee in Research under protocol number CAAE: 03996612.3.0000.5053.

The inclusion criteria were: pregnant women with PTL-related complaints, at GAs between 24 and 34 weeks, with cervix dilation ≤ 2 cm, and voluntarily acceptance to participate in the study upon signing the free and informed consent term (all lengths of uterine cervix were included).

The exclusion criteria were: patients with transvaginal bleeding, broken amniotic sac, twin gestation, with reduced cognition/consciousness, and those who underwent vaginal touch, used vaginal medications or douche, and had sexual intercourse in the 24 hours prior to study selection.

Initially, the endocervical material was collected from participants using only a sterile swab; these samples were used to detect fibronectin through the fFN rapid test. The presence of fibronectin in the cervical sample was determined qualitatively. The vaginal touch would only be performed after the collection of endocervical material. Subsequently, the MUCL was performed by TVUS. The following criteria were adopted to ensure uniformity in this measurement: the internal cervical orifice should be clearly visible with a gentle depression as an isosceles or funnel triangle; the entire length of the cervical canal should be clearly visible; the external cervical orifice should be visible symmetrically; the external cervix surface should be clearly identified; the endocervical funnel, if present, was not included in the MUCL.

All cervix measurements were performed by the same examiner as well as the sample collections for the fFN test. The cervix was determined as short when the length was under 25 mm. Other data were collected through an interview based on a form with predefined questions addressing socioeconomic, reproductive, and clinical-obstetric information. The follow-up until delivery of outcomes on data annotation was conducted through telephone contact with these pregnant women.

Data analysis was performed through the Epi-info software. The relative risk (RR) was calculated for each parameter with 95% confidence interval (95% CI) and a significance level of 5% (*p* < 0.05) in all tests. The risk assessment for PTD and testing for positive fFN, PTD and MUCL, and risk assessment when both variables were positive were all evaluated independently and included in the analysis. In addition, the risk for evolution to childbirth was evaluated at 48 hours, 7 days, and 14 days when the fFN and MUCL tests were each positive, separately or together.

## Results

A total of 53 pregnant women were included in the analyses. The mean age was 22.80 years, 60.38% lived in urban areas, 52.83% had completed middle school education, and 62.26% had a monthly income of up to 1 Brazilian minimum wage. The obstetric history showed that 56.60% of the patients were primiparous. Out of the non-primiparous patients, only 21.74% had a history of previous PTL. The mean GA at study admission was 31.83 weeks (Table 1).

**Table 1:** Characteristics of the analyzed sample

**Average age (years)**		22.8 *(SD 7.32)*
**Origin**		
Urban		60.38%
Rural		39.62%
**Family income (%)**		
Up to one minimum wage		62.26%
More than one minimum wage		37.74%
**Education (%)**		
Illiterate		3.77%
Middle school level		52.83%
High school level		39.62%
College level		3.77%
**Previous pregnancies (%)**		
Primiparous		56.60%
Non-primiparous		43.40%
**Previous preterm delivery (%)**		
Yes		21.74%
No		78.26%
**Gestational age at study admission (average weeks)**		Average: 31.83 *(SD 2.03)*
**Patients who received tocolysis (Nifedipine)**		88.68%
**MUCL by TVUTS**		Average: 21.7mm *(SD 8.9)*
	Cervix < 25mm	58.49%
	Cervix ≥ 25mm	41.51%
**fFN rapid test results**	Positive	41.51%
	Negative	58.49%

Abbreviations: fFN, fetal fibronectin; MUCL by TVUTS, measurement of uterine cervix length by transvaginal ultrasonography; SD, standard deviation.

Almost all pregnant women received tocolysis when hospitalized (88.68%), but when crossing that variable with preterm birth, we did not find a difference of risk (RR = 1.39, 95% CI, 0.46–4.21, *p* = 0.41).

The MUCL mean value measured by TVUS was 21.7 mm with a standard deviation of 0.89 and 75^th^ percentile at 28.0 mm. The cut-off value of 25 mm used to transform this variable into a qualitative one showed that 58.49% of the participants had MUCL shorter than 25 mm at the time of study admission.

A total of 41.51% of participants showed positive fFN rapid-test results during study admission (Table 1).

Out of the 53 patients initially analyzed, 48 (90.57%) were followed-up until delivery; of these, 26 delivered before 37 weeks (54.17%).

The comparison between time of delivery and MUCL showed a tendency of increased risk of PTL when MUCL was shorter than 25 mm, however, without statistical significance (RR: 1.83, 95% CI, 0.99–3.36, *p* = 0.09). Nevertheless, a statistical difference was observed between the meantime in weeks from study admission to delivery (2.98 × 5.00, *p* = 0.03) (Table 2).

**Table 2:** Relation between measurement of the uterine cervix, fetal fibronectin rapid test results, and the outcome of preterm delivery

**MUCL < 25 mm X PTD**			
Relative risk	1.83	95% CI 0.99–3.36	*p* = 0.09^a^
Mean time between testing and delivery			
	MUCL < 25 mm	2.98 weeks	*p* = 0,03^b^
	MUCL ≥ 25 mm	5.00 weeks	
**fFN rapid test X PTD**			
Relative risk	3.5	95% CI 1.39–8.79	*p* = 0.002^a^
Mean time between testing and delivery			
	Positive fFN	1.94 weeks	*p* = 0.0003^b^
	Negative fFN	5.20 weeks	
**Both tests X PTD**			
Relative risk	2.70	95% CI 1.08–6.72	*p* = 0.002^a^
Mean time between testing and delivery			2.17 weeks

Abbreviations: CI, confidence interval; fFN, fetal fibronectin; MUCL, measurement of uterine cervix length; PTD, preterm delivery.

*p*:p-value. Statical tests: a-*X*^2^; b-*T test.*

The association of positive fFN rapid-test results with PTL was statistically significant (RR: 3.50; 95% CI: 1.39–8.79; *p* = 0.002); the same occurred in relation to the mean time in weeks from study admission to delivery (1.94 × 5.20, *p* = 0.0003) (Table 2).

The risk for PTL was also increased when both results (fFN and MUCL) were used; however, this risk was lower than that considering the fFN rapid test alone (RR: 2.70; 95% CI 1.08–6.72; *p* = 0.002) as demonstrated by the average number of weeks from study admission to delivery (2.17) (Table 2).

The MUCL results showed that the relative risk of PTL is RR = 1.30, 95% CI 1.02–1.67, and *p* = 0.11 in the first 48 hours; RR = 1.43, 95% CI % 0.99–2.06, and *p* = 0.12 in up to 7 days; and RR = 2.03, 95% CI 1.26–3.27, and *p* = 0.008 in up to 14 days (Table 3).

**Table 3:** Risk for preterm delivery outcome in 48 hours and 7 and 14 days relative to measurement of the uterine cervix and fetal fibronectin results

Evolution to PTD			
	Within 48 hours	In up to 7 days	In up to 14 days
MUCL < 25 mm	F = 16.67%	F = 25.00%	F = 35.41%
	RR = 1.30	RR = 1.43	RR = 2.03
	*p* = 0.11	*p* = 0.12	*p* = 0.008
	95% CI 1.02–1.67	95% CI % 0.99–2.06	95% CI 1.26–3.27
Positive fFN	F = 18.75%	F = 29.17%	F = 33.33%
	RR = 1.75	RR = 2.88	RR = 3.57
	*p* = 0.0006	*p* = 0.0001	*p* = 0.0002
	95% CI 1.20–2.53	95% CI, 1.57–5.31	95% CI 1.63–7.81
MUCL < 25 mm & positive fFN	F = 16.67%	F = 22.91%	F = 27.08%
	RR = 1.74	RR = 2.22	RR = 2.76
	*p* = 0.0001	*p* = 0.0001	*p* = 0.0002
	95% CI 1.14–2.64	95% CI 1.22–4.04	95% CI 1.27–5.96

Abbreviations: CI, confidence interval; F, frequency; fFN, fetal fibronectin; MUCL, measurement of uterine cervix length; *p*, p value; PTD, preterm delivery; RR, relative risk.

Statistical test: *X* ^2^.

When the fFN rapid test was positive, the relative risk for PTL in 48 hours was RR = 1.75, 95% CI 1.20–2.53, and *p* = 0.006; RR = 2.88, 95% CI, 1.57–5.31, and *p* = 0.0001 in up to 7 days; and RR = 3.57, 95% CI 1.63–7.81, and *p* = 0.0002 in up to 14 days (Table 3).

A significant association with PTL was observed in all scenarios when evaluating the positivity of both tests (MCCU < 25 mm and positive fFN); the relative risk for PTL was RR = 1.74, 95% CI 1.14–2.64; and *p* = 0.0001 in 48 hours; RR = 2.22, 95% CI 1.22–4.04, *p* = 0.0001 in up to 7 days; and RR = 2.76, 95% CI 1.27–5.96, and *p* = 0.0002 in up to 14 days (Table 3).

## Discussion

Although digital uterine cervix evaluation is part of the routine examination of patients who are at high risk for prematurity, this method is not often a safe way to recognize early cervical alterations. Yamasaki et al^13^ verified that the MUCL by TVUS showed a better accuracy for PTL diagnosis than vaginal touch in pregnant women at high risk because the portion above the anterior fornix can be evaluated through TVUS but not through vaginal touch.

In this study, considering only the cervices < 25mm, there is no statistically significant increase of risk of PTD, but the interval of time between the measurement and the delivery was statistically different, two weeks longer in pregnant women when the MUCL > 25mm. In 1996, Iams et al^14^ reported that MUCL under 25 mm indicated a PTD positive predictive value of 17.8% and negative of 97%. Therefore, the MUCL helps distinguishing pregnant women from false PTL, which may prevent unnecessary interventions. Likewise, a pregnant woman with short MUCL deserves extended attention through the performance of additional tests using antenatal corticoid and preventive measures.

Tanvir et al (2014),^15^ evaluated the MUCL in 130 pregnant women between 22 and 24 weeks of GA using the length < 25 mm as the cut-off point. That study reports that among the patients with short MUCL (16 women), 13 evolved to PTD, thus demonstrating the importance of this measurement as a PTD predicting factor.^15^ To et al (2001)^16^ demonstrated the inverse relationship between PTD risk and MUCL; the risk can reach 78% when the length is shorter than 5 mm, decreases to 4% when the length is up to 15 mm, and decreases to 0.5% when the length is greater than 50 mm in asymptomatic patients. Our study highlighted a similar trend; the difference in the magnitude of the reduction may be due to the small sample, did not allow our results to reach statistical significance.

Fetal fibronectin is an extracellular matrix glycoprotein that is produced by amniocytes and cytotrophoblasts and has been shown to predict spontaneous preterm birth.^17^ Our study observed the use of the qualitative fFN test showed an increase of PTD isolated or in association with MUCL, and when the test was negative, the time between to delivery was greater than three weeks, showing superior to MUCL. Similar results in a multicenter study, Brujin et al (2016),^18^ demonstrated the comparison between the quantitative and qualitative results of fFN tests associated with MUCL and the risk for PTD in seven days. These authors showed that the quantitative fFN test presents the same accuracy compared with the qualitative fFN test associated with MUCL. However, the association between MUCL and the qualitative measure of fFN shows an advantageous capacity to predict PTD.^18^ Other studies demonstrated that the use of the qualitative fFN test alone was not enough to increase the prediction of PTD compared with the use of the fFN test in association with MUCL.^19^ ^20^ Deshpande et al (2013),^21^ evaluated the cost-effectiveness of using the rapid fibronectin test in symptomatic pregnant women and reported that the test, used in isolation, had moderate accuracy and could identify patients with negative test results who would not need intervention. Magro-Malosso et al (2017),^22^ observed that positive fFN tests were detected in 33% of symptomatic patients and significantly associated with PTD at 34 weeks and within 48 hours, 7, 14, and 21 days after admission (*p* < 0.05); this association was not observed in the asymptomatic group. This study showed an increased risk of PTD, mainly when analyzed the use of fFN test and both tests. The MUCL increased the risk of PTD only within 14 weeks. Van Baaren et al (2013)^23^ concluded that the best cost-benefit ratio is in the combined application of the fFN test and MUCL. Hadži-Legal et al (2016)^24^ reported similar results in symptomatic pregnant women observing that the combination of these tests turned out to be an excellent predictor of PTD within 14 days of admission.^24^ Despite the similarities to our results, these authors state that there are no high-quality studies about the evaluation of this test, reducing the magnitude of our results.

In another study with a small sample of 30 patients, the fFN test used in this sample was not shown to be predictive enough to inform the decisions of clinicians and pregnant mothers to delay evacuation to a regional birthing center.^25^ Similar results in systematic review and meta-analysis of randomized clinical trials the fFN testing in singleton gestations with threatened preterm labor is not associated with the prevention of preterm birth or improvement in perinatal outcome but is associated with higher costs.^17^ The different trends shown by our results may be due to the small sample and a distinct population group of Brazilian women not included in these studies.

Our study has several strengths, the main one being that it is the first study in the Northeast of Brazil that associates fFN e MUCL in PTD, and some weaknesses, including the facts that this study is analytical and non-interventional and has a small sample.

## Conclusion

In symptomatic pregnant women, we conclude that the MUCL < 25 mm and positive fFN rapid test indicate increased risk for PTD. Further studies with larger sample sizes could contribute in supporting the results presented in the current study.
